# Mr. Custance, on Quackery

**Published:** 1800-07

**Authors:** Geo. Custance

**Affiliations:** Kidderminster


### *To the Editors of the Medical and Phyſical Journal.*

GENTLEMEN,

IT muſt give great pleaſure to every lover of, and well-wiſher to, the ſcience of medicine, to ſee that ſeveral of your Correſpondents have called the attention of the profeſſion to the ſubject of Quackery; which is likely to become more extenſively pernicious, from the public teſtimony which a few perſons in exalted ſtations have thought it incumbent upon them to bear to the efficacy of certain advertiſed infallible remedies. The danger obviouſly reſulting from Empiriciſm will be readily admitted by all who have been regularly educated, and are converſant with the powers of ſimple and compound medicines, and the œconomy of the human ſyſtem; but it is not ſo eaſy to diſcover a method of checking the public progreſs of this evil. The plan which your correſpondent, Aliquis, propoſes of referring the ſubject to Parliament, would certainly be effectual, could an act be paſſed, forbidding the *advertizing* any kind of noſtrum in any news-paper, hand-bill, or in any other way whatever; for it is by theſe means that the public attention is conſtantly directed to the proprietors, whoſe names would ſoon fall into contempt, if their ſkill and reputation were analyzed by the moſt proper teſt — the number of *real* cures which they wrought. So well aware of this fact are theſe infallible doctors themſelves, that they find it neceſſary to vary the form of their advertiſements, to obtain even *them* a reading.

Mr. Bartley has, with probable ſucceſs, (in No. XV.) brought into view ſome facts, to prove the danger, expence, and folly which thoſe run into, who are the dupes of Quackery. It appears to me, that the frequent expoſure of ſuch facts would have the beſt effect: but in order that they might, it is neceſſary they ſhould be circulated through the ſame channel as the doctors' advertiſements; for the readers of your excellent Journal are not the perſons likely to be hurt by the ignorant and preſumptuous empyric. It is, generally ſpeaking, the middling and lower ranks of people who are the ſubjects and dupes of this impoſture: And that the public may have the better opinion of the medicines, the proprietors commonly inform them, that, "*by the bleſſing of God,*" they undertake, with their powders, potions, or balſams, to cure all diſorders. Thus ignorance and blaſphemy unite in picking the pockets and ruining the conſtitutions of thouſands. It might be well if the following general obſervation, amongſt others that might be mentioned, could any way be impreſſed upon the public mind: —

The pretenſion to infallibility in any one medicine, in curing any one diſorder, is abſurd; much more ridiculous is it to ſuppoſe, that any medicine will remove *all kinds* of complaints: for every medicine poſſeſſes active properties, or it does not. If it be active, it muſt be dangerous to apply it, indiſcriminately, to perſons of every age, and without regard to their habits of living. Such an active medicine may be very efficacious in ſtrengthening a debilitated conſtitution, but would be highly injurious if exhibited for acute rheumatiſm, or any other inflammatory diſorder, and, *vice versa*; conſequently, it is unwiſe and unſafe to apply the ſame remedy in all caſes. Should the medicine be inactive (which is doubtleſs often the caſe, or more miſchief muſt follow) it can be of no other utility than to work upon the imagination; thereby amuſing the patient, while his pockets are rifled, unperceived.

It is to be lamented, that while many are robbed of their money and health by mercenary and rapacious impoſtors, not a few are led into error and danger by the noſtrums of thoſe who are actuated by the pureſt motives in recommending them to the public, through the channel of various periodical publications. I allude now particularly to the circumſtances of balm, barm, or yeaſt, having been, in the moſt unqualified manner, held out as a certain cure in *putrid fevers*. That which is ſimple in its nature, and eaſy to be come at, is ſure to go down with the multitude. It is not denied, that the fixed air in the balm may occaſionally have ſome good effect in putrid diſeaſes; but I condemn the conduct of thoſe who, in contending with ſo dangerous and powerful an enemy as typhus fever, place yeaſt in the front and heat of the battle, whilſt bark, wine, and opium are put in the rear, or, perhaps, ſtationed at a diſtance as a "corps de reſerve."

In the Armenian Magazine for Auguſt laſt, a very brilliant diſplay is exhibited of the wonderful effects of this wonderful medicine. Amongſt other caſes, "too tedious to mention," a man is ſtated to have been "in the laſt ſtage of putrid fever," when the yeaſt was given, "diluted with water," and the "next morning he was apparently well, *and walking in his garden*." So in the Monthly Magazine for laſt November, it is aſſerted, that "a ſtrong Dutch girl, brought low with a putrid fever, was ordered ſome yeaſt, and was *immediately* cured." I have frequent opportunities of ſeeing and treating putrid fevers, but have never witneſſed theſe ſudden recoveries from the laſt ſtage of them to "walking about in gardens." But as people in general are ignorant of the diſorder, no wonder if, being ill judges of the moſt ſuitable remedies, they throw throw out to a ſinking friend even a rope of ſand, that is handed to them by a kind but miſtaken neighbour.

I lately attended the ſervant of a gentleman, in the laſt ſtage of typhus; pulſe 140; ſubſultus tendinum, &c. I informed him, I thought the caſe a dangerous one. He ſaid, he wiſhed me to try yeaſt, as he had ſeen it ſtrongly recommended in the Monthly Magazine. I told him that, to give it a fair trial, I ſhould lay aſide every thing elſe. I did ſo; and the ſecond ſpoonful of yeaſt brought on ſuch a purging as had well nigh carried her off; but by returning to opium, wine, and bark, ſhe happily recovered. About a week after, another young woman, who had been ill nine days, and was covered with petechiæ, and even vibices, was directed by her maſter to take the yeaſt; and if ſhe was no better in the morning, to ſend for me. In about four hours after ſhe took the yeaſt, a violent purging came on, which I could not check, and ſhe died the next day. There were five others in the family, who were all ſucceſſively attacked, and to whom I exhibited opium and volatile alkali pretty plentifully; they all recovered excepting an infant two months old, to whom it was impoſſible to give any thing. Thus we ſee, that however well "a ſtrong Dutch girl" may bear *working* with yeaſt, it will not be ſafe to have recourſe to ſuch means in recovering our fair country-women from putrid fevers. The publiſher of this noſtrum, in the Armenian Magazine, tells us, he was firſt led to this idea from the fact that a piece of putrid meat is ſweetened by being ſuſpended over working beer. But to conclude from hence, that yeaſt (that is, the fixed air in it) will correct the putridity of the blood, and other animal circulating fluids, is fallacious; for it is not generally admitted that ſuch putridity actually exiſts in the blood: But ſuppoſe it does; if we allow ourſelves to judge of the effects of any ſubſtance on the living fibre and ſyſtem, from its known action on *dead* matter, we ſhall be led into the moſt dangerous miſtakes. The concentrated vitriolic acid preſerves dead animal matter from putrefaction; but we know that it cannot be ſwallowed with ſafety, except in ſmall quantities and diluted with water. So, on the other hand, humidity is known to promote the putrefaction of dead animals, while water may be ſwallowed, and externally applied, not only with ſafety, but even with advantage, in putrid fevers. Upon the whole then, it is plain, that unleſs the public in general were well acquainted with the various diſeaſes incident to mankind, it muſt be dangerous to commit the treatment of them to ſuch unſkilful hands. We do not act ſo in the other profeſſions. Does not the client employ counſel learned in the law to plead his cauſe? Will a wiſe government entruſt the command of its armies and navies to men unacquainted with military and naval tactics?

Permit me to embrace this opportunity of tranfmitting to you two cafes of *Croup* fuccefsfully treated with the Digitalis, which, I think, promifes to be a very efficacious remedy in that dreadful and generally fatal diforder. I fhould have waited to fee its effects in a greater number of cafes, before I had fent you the refult, but am defirous of throwing out the hint to practitioners in general as early as poffible, and fhall be happy to fee it has been improved upon with the defired fuccefs in their future treatment of the croup.

Mary Bell, four years of age, was brought to me about twenty-four hours after being attacked with the ufual fymptoms of croup. The hoarfenefs, fhrill voice, and dyfpnoea were very confiderable. I ordered five drops of tinct. digitalis to be given her in water every four hours; and the next day fhe was quite free from the complaint, which never returned.

Mary Millard, a year and half old, was attacked on the 11th of this month with hoarfenefs, a barking cough, and great dyfpnœa. I faw her about twenty hours after the firft appearance of thefe fymptoms, and found her extremely reftlefs, with a very quick pulfe. I ordered five drops of the tinct. digitalis every four hours. 12th, Symptoms relieved; p. ftill quick; has had one ftool. Increafed the dofe to fix drops:—at night p. lefs frequent; three ftools; hoarfenefs and barking almoft gone. 13th, Still fome barking, but coughs lefs frequently:—8 o'clock at night, very reftlefs; hoarfenefs and dyfpnœa increafed; p. very quick. Cont. tinctura. 14th, Slept well; frequent ftools; dyfpnœa and hoarfenefs much relieved. Adde tinct. opii gt. ij. fing. dos. digitalis. 8 o'clock at night, five ftools fince morning; p. much lefs frequent; dyfpnœa and hoarfenefs ftill better. 15th, A good night; two ftools; pulfe calm; dyfpnœa quite gone; fome hoarfenefs remains. 16th, Symptoms of the croup quite gone. 21ft, Continues free from complaint. I am,

Gentlemen,

Your moft obedient fervant,

GEO. CUSTANCE.

*Kidderminfter,*

*May* 22, 1800.

